# Prognostic benefit of surgical management in renal cell carcinoma patients with thrombus extending to the renal vein and inferior vena cava: 17-year experience at a single center

**DOI:** 10.1186/1471-2490-13-47

**Published:** 2013-10-14

**Authors:** Shingo Hatakeyama, Takahiro Yoneyama, Itsuto Hamano, Hiromi Murasawa, Takuma Narita, Masaaki Oikawa, Kazuhisa Hagiwara, Daisuke Noro, Toshikazu Tanaka, Yoshimi Tanaka, Yasuhiro Hashimoto, Takuya Koie, Chikara Ohyama

**Affiliations:** 1 Department of Urology, Hirosaki University Graduate School of Medicine, 5 Zaifu-chou, 036-8562 Hirosaki, Japan; 2 Department of Advanced Transplant and Regenerative Medicine, Hirosaki University Graduate School of Medicine, Hirosaki, Japan

**Keywords:** Renal cell carcinoma, Radical nephrectomy with thrombectomy, Tumor thrombus, Prognostic factors

## Abstract

**Background:**

Management of renal cell carcinoma (RCC) with tumor thrombus extending to the renal vein and inferior vena cava (IVC) is challenging. The aim of this study was to evaluate the benefit of surgical management in such patients.

**Methods:**

From February 1995 to February 2013, 520 patients were treated for RCC at Hirosaki University Hospital, Hirosaki, Japan. The RCC patients with tumor thrombus extending to the renal vein (n = 42) and IVC (n = 43) were included in this study. The records of these 85 patients were retrospectively reviewed to assess the relevant clinical and pathological variables and survival. Prognostic factors were identified by multivariate analysis. The benefit of surgical management was evaluated using propensity score matching to compare overall survival between patients who received surgical management and those who did not.

**Results:**

RCC was confirmed by pathological examination of surgical or biopsy specimens in 74 of the 85 patients (87%). Sixty-five patients (76%) received surgical management (radical nephrectomy with thrombectomy). Distant metastasis was identified in 45 patients (53%). The proportion of patients with tumor thrombus level 0 (renal vein only), I, II, III, and IV was 49%, 13%, 18%, 14%, and 5%, respectively. The estimated 5-year overall survival rate was 70% in patients with thrombus extending to the renal vein and 23% in patients with thrombus extending to the IVC. Multivariate analysis identified thrombus extending to the IVC, presence of distant metastasis, surgical management, serum albumin concentration, serum choline esterase concentration, neutrophil-lymphocyte ratio, and Carlson comorbidity index as independent prognostic factors. In propensity score-matched patients, overall survival was significantly longer in those who received surgical management than those who did not.

**Conclusions:**

Surgical management may improve the prognosis of RCC patients with thrombus extending to the renal vein and IVC.

## Background

In renal cell carcinoma (RCC), thrombus extends to the inferior vena cava (IVC) in 4–15% of patients [1,2]. With recent advances in surgical techniques and instruments, radical nephrectomy with thrombectomy has been reported to improve the prognosis of RCC patients without distant metastasis [3]. However, surgery carries significant risks of perioperative morbidity and mortality [4]. Previous studies reported perioperative mortality rates of 0.1% for general surgery patients overall and 5–10% for patients undergoing radical nephrectomy with IVC thrombectomy [5-7]. Although several studies reported large series of RCC patients who underwent radical nephrectomy with thrombectomy [8-10], only a few studies compared outcomes between patients who underwent surgery and those who did not [8,11]. The role of nephrectomy with thrombectomy in patients with distant metastasis also remains unclear. In this study of RCC patients with renal vein and IVC thrombus, we compared outcomes between patients who received surgical management and those who did not.

## Methods

### Patients and staging

This study was performed in accordance with the ethical standards of the Declaration of Helsinki. The study protocol was approved by the institutional review board of Hirosaki University School of Medicine. A total of 520 RCC patients were treated in our clinic from February 1995 to February 2013. Of these, 42 patients had tumor thrombus extending to the renal vein (RV thrombus group) and 43 had tumor thrombus extending to the IVC (IVC thrombus group). The records of these 85 patients were retrospectively reviewed to assess the relevant clinical and pathological variables and survival.

All 85 patients underwent routine preoperative blood tests; brain, chest, and abdominal computed tomography (CT); abdominal magnetic resonance imaging; and/or bone scintigraphy. Gross extension of tumor thrombus into the venous system was detected by preoperative radiological examinations, including contrast-enhanced CT, magnetic resonance imaging, and/or vena cavography.

**Figure 1:**
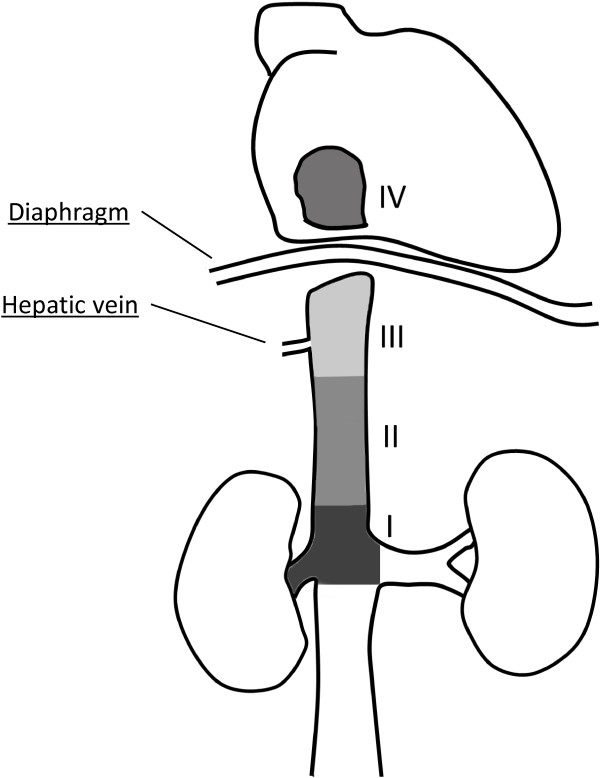
**Classification of tumor thrombus level according to the Mayo staging system.** Level 0, thrombus extending to the renal vein; level I, thrombus extending into the IVC to no more than 2 cm above the renal vein; level II, thrombus extending into the IVC to more than 2 cm above the renal vein but not to the hepatic vein; level III, thrombus extending into the IVC to above the hepatic vein but not to the diaphragm; and level IV, thrombus extending into the supradiaphragmatic IVC or right atrium.

The level of tumor thrombus was determined according to the Mayo classification (Figure 1): level 0, thrombus extending to the renal vein only; level I, thrombus extending into the IVC to no more than 2 cm above the renal vein; level II, thrombus extending into the IVC to more than 2 cm above the renal vein but not to the hepatic vein; level III, thrombus extending into the IVC to above the hepatic vein but not to the diaphragm; and level IV, thrombus extending into the supradiaphragmatic IVC or right atrium [12]. Pathological diagnoses were determined according to the 2009 Union for International Cancer Control/American Joint Committee on Cancer TNM system [13,14]. Overall patient condition was assessed using the Eastern Cooperative Oncology Group performance status (ECOG-PS) [15].

The basic treatment strategy for RCC with tumor thrombus was surgical extirpation of the tumor, with the aim of prolonging survival. Patients received non-surgical management if they refused surgery or if they had worsening systemic comorbidities, ECOG-PS >3, extremely advanced metastatic disease that would be difficult to control, or severe complications.

### Surgical management

All patients who received surgical management underwent radical nephrectomy, thrombectomy, and lymph node dissection. Surgery was performed via a flank or midline abdominal incision, depending on surgeon preference and the characteristics of the tumor and associated thrombus. In patients with supradiaphragmatic IVC thrombus, the liver was mobilized to expose the retrohepatic vena cava by incision of the falciform, triangular, and coronary ligaments, in cooperation with the Department of Gastroenterological Surgery. In patients with right atrium thrombus, sternotomy and extracorporeal circulation with cardiopulmonary bypass were performed in cooperation with the Department of Cardiothoracic Surgery.

### Follow-up schedule

Patients were evaluated for postoperative recurrence and general condition by blood count, blood biochemistry analysis, and chest and abdominal CT every 3 months for the first year, and every 6 months thereafter. Chest CT was used instead of chest X-ray in consideration of the relative risks and benefits of these examinations. Brain CT was performed when any new metastatic lesions or neurological symptoms were observed. Patients did not receive routine postoperative adjuvant therapy, but additional treatment was given when new metastatic lesions were identified.

### Statistical analysis

Patients in the RV thrombus group and IVC thrombus group were analyzed separately. To evaluate the benefit of surgical management, we compared overall survival between propensity score-matched patients (difference in score ≤0.03) who received surgical management and those who did not. Propensity scores were calculated by logistic analysis using conventionally recognized risk factors for survival at the time of the initial visit, including age, sex, ECOG-PS, level of tumor thrombus, presence of distant metastasis, and Charlson comorbidity index.

Survival was calculated using the Kaplan–Meier method. The significance of differences between groups was evaluated using the log-rank test. Variables were compared between groups using the Student’s *t-*test or Mann–Whitney *U* test. Prognostic factors were identified by univariate and multivariate analyses using the Cox proportional hazards model, and hazard ratios (HRs) with 95% confidence intervals were calculated. All statistical analyses were performed using the SPSS software package version 19.0 (SPSS, Chicago, IL, USA) and GraphPad Prism version 5.03 (GraphPad Software, San Diego, CA, USA). A value of P < 0.05 was considered statistically significant.

## Results

**Figure 2:**
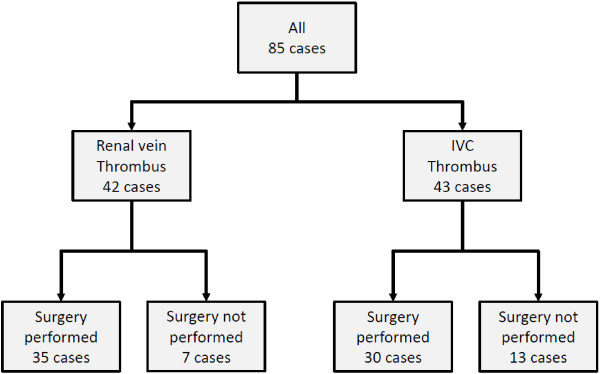
**Management of enrolled patients.** A total of 85 patients were enrolled in this study, including 42 in the RV group and 43 in the IVC group. Sixty-five patients underwent radical nephrectomy with thrombectomy and 20 did not undergo surgery.

Patient characteristics are shown in Table 1 and overall survival is shown in Figure 2. The diagnosis of RCC was confirmed by pathological examination of surgical or biopsy specimens in 74/85 patients (87%) and by imaging examination findings in 11 patients (13%). Forty-two patients (49%) had thrombus extending to the renal vein and 43 (51%) had thrombus extending to the IVC. In patients with IVC thrombus, the thrombus was classified as level I, II, III, and IV in 11 patients (13%), 15 patients (18%), 12 patients (14%), and 5 patients (6%), respectively. The prevalence of multiple organ metastases in surgical and non-surgical treatment group was 6% and 57% in renal vein, 14% and 50% in level I, 15% and 100% in level II, 25% and 50% in level III, 0% and 67% in level IV, respectively.

**Table 1:** Patient characteristics

		**Renal vein**			**IVC thrombus**		
	**All**	**Surgery performed**	**Surgery not performed**	***P value***	**Surgery performed**	**Surgery not performed**	***P value***
n	85	35	7		30	13	
Age*	62 ± 12	63 ± 10	62 ± 10	*0.790*	59 ± 14	37 ± 11	*0.078*
Gender		24/11	4/3	*0.558*	17/13	10/3	*0.021*
ECOG-PS	0.6 ± 1.1	0.3 ± 0.7	0.7 ± 1.5	*0.484*	0.5 ± 1.0	1.6 ± 1.4	*0.021*
Radical surgery	65(76%)	35	0		30	0	
Perioperative morality within 1 month		0(0%)			2(7%)		
Thrombus level							
Renal vein	42(49%)	35	7				
I	11(13%)				7(23%)	4(31%)	*0.203*
II	15(18%)				13(43%)	2(15%)	
III	12(14%)				8(27%)	4(31%)	
IV	5(6%)				2(7%)	3(23%)	
Clinical TNM							
cT3	78(92%)	35(100%)	6(86%)	*0.024*	30(100%)	7(54%)	*0.000*
cT4	7(8%)	0(0%)	1(14%)		0(0%)	6(46%)	
cN+	20(24%)	1(3%)	5(71%)	*<0.0001*	5(17%)	9(69%)	*0.001*
cM+	45(53%)	11(31%)	6(86%)	*0.008*	17(33%)	11(85%)	*0.041*
Multiple organ metastasis	19(22%)	2(6%)	4(57%)	*0.006*	5(17%)	8(62%)	*0.041*
Thrombus level (I/II/III/IV)					1/2/2/0	2/2/2/2	
Histology							
Clear	67(79%)	31(89%)	5(71%)	*0.426*	27(93%)	4(31%)	*0.508*
Others	7(8%)	4(11%)	0(0%)		3(7%)	0(0%)	
Unknown	11(13%)	0(0%)	2(29%)		0(0%)	9(96%)	
Grade							
G1/2	43(51%)	25(71%)	0(0%)	*0.002*	12(40%)	4(31%)	*0.024*
G3	31(36%)	10(29%)	5(71%)		12(40%)	4(31%)	
Unknown	11(13%)	0(0%)	2(29%)		0(0%)	9(69%)	
Hemoglobin (g/dl)*	11.6 ± 2.4	12.0 ± 2.2	104 ± 2.0	*0.081*	11.4 ± 2.3	11.2 ± 3.3	*0.752*
Neutrophil-lymphocyte ratio*	3.1 ± 1.5	2.8 ± 1.2	3.6 ± 1.2	*0.156*	3.1 ± 1.9	3.6 ± 1.5	*0.402*
Serum albumin (g/dl)*	3.8 ± 0.6	3.9 ± 0.6	3.4 ± 0.8	*0.122*	3.9 ± 0.6	3.5 ± 1.5	*0.080*
Renal function (ml/min./1.73 m^2^)*	63 ± 23	71 ± 20	52 ± 21	*0.063*	60 ± 23	56 ± 25	*0.605*
Lactate dehydrogenase (LDH,|IU/L)*	214 ± 112	185 ± 45	304 ± 217	*0.198*	199 ± 80	274 ± 172	*0.154*
Choline esterase (U/L)*	224 ± 105	248 ± 98	234 ± 72	*0.665*	215 ± 130	172 ± 102	*0.252*
Serum sodium (mEq/L)*	141 ± 2.5	142 ± 2.3	139 ± 3.7	*0.111*	142 ± 2.2	139 ± 2.3	*0.005*
Correlated calcium (mg/dL)*	9.6 ± 0.8	9.4 ± 0.6	9.9 ± 0.8	*0.153*	9.6 ± 0.8	10.1 ± 0.8	*0.069*
C-reactive protein (CRP, mg/dL)*	4.4 ± 5.4	4.1 ± 6.1	4.8 ± 3.3	*0.648*	3.7 ± 6.0	6.4 ± 4.6	*0.125*
Charlson comorbidity index*	7.7 ± 3.4	6.6 ± 3.1	11.3 ± 1.6	*<0.0001*	7.2 ± 3.1	10.3 ± 3.0	*0.004*
Molecular targeted agents	18(21%)	5(14%)	4(57%)	*0.012*	6(20%)	3(23%)	*0.549*
Deceased	43(51%)	8(23%)	4(57%)	*0.067*	20(67%)	11(85%)	*0.228*

*Mean ± SD.

**Figure 3:**
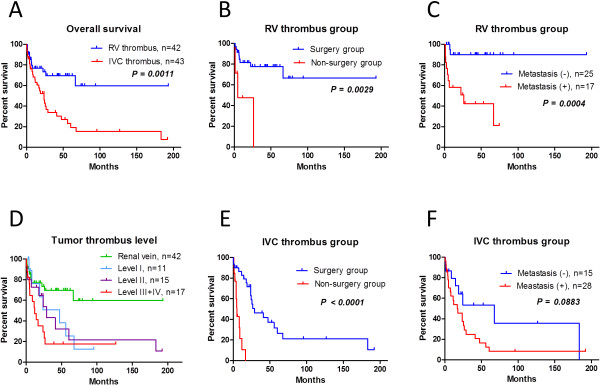
**Survival in the RV and IVC thrombus groups, according to surgical management and distant metastasis. (A)** Overall survival in the RV thrombus and IVC thrombus groups. **(B)** In the RV thrombus group, survival was significantly longer in patients who received surgical management than those who did not. **(C)** In the RV thrombus group, distant metastasis was a powerful prognostic factor. **(D)** In the IVC thrombus group, thrombus level was not significantly correlated with overall survival. **(E)** In the IVC thrombus group, survival was significantly longer in patients who received surgical management than those who did not. **(F)** In the IVC thrombus group, distant metastasis was not a significant prognostic factor.

Sixty-five patients (76%) underwent radical nephrectomy with thrombectomy, and 20 did not receive surgical management. None of the patients who received surgical management underwent preoperative renal artery embolization. The median follow-up period was 26 months in patients who received surgical management and 5 months in patients who did not. Among the patients who did not receive surgical management, eight received immunotherapy or interferon-α 6,000,000 IU three times/week, seven received molecular targeted therapy, one underwent tumor embolization, and four received best supportive care only. The reason for non-surgical management was multiple organ or unresectable metastasis in 14 patients (lung and lymph nodes, n = 6; lung and bone, n = 2; lung, n = 2; lung, bone, and brain, n = 1; lung and liver in a patient with duodenal invasion, n = 1; brain, n = 1; lymph nodes, n = 1), patient refusal in 4 patients, dementia in 1 patient, and ECOG-PS >3 in 1 patient. In the whole group of 85 patients, the estimated median overall survival time was 41 months and the estimated 5-year overall survival rate was 40% (Figure 3A, Table 2). At the time of this report, 43 patients (51%) had died of their disease, including 24 (43%) who received surgical management and 15 (75%) who did not (P = 0.003). In all patients who did not receive surgical management, the main cause of death was cachexia. In patients who received surgical management, the estimated median survival time was 60 months and the estimated 5-year overall survival rate was 54%. In patients who did not receive surgical management, the estimated median survival time was 8.2 months and the estimated 5-year survival rate was 0% (Table 2). Distant metastasis was present at the time of diagnosis in 45 patients (53%). In patients with distant metastasis at presentation, the median overall survival time was 11 months and the estimated 5-year survival rate was 21%. In patients without distant metastasis at presentation, the estimated median survival time was 24 months and the estimated 5-year survival rate was 80% (Table 2). The independent prognostic factors identified by multivariate analysis using the Cox proportional hazards model were thrombus level, presence of distant metastasis, surgical management, serum albumin concentration, serum choline esterase concentration, neutrophil-lymphocyte ratio, and Charlson comorbidity index (Table 3).

**Table 2:** Estimated median overall survival times and 5-year overall survival rates

	**All patients**	**Surgery group**	**Non-surgery group**	**Without metastasis**	**With metastasis**
Median survival (M)	41	60	8.2	24	11
5-year survival (%)	40	54	0	80	21
	RV thrombus				
Median survival (M)	Undefined	Undefined	49	Undefined	26
5-year survival (%)	70	78	0	90	42
	IVC thrombus				
Median survival (M)	24	29	5.1	68	17
5-year survival (%)	23	32	0	54	12

**Table 3:** Univariate and multivariate analyses for overall survival

	**All**						**RV thrombus group**						**IVC thrombus group**					
	**Univariate**			**Multivariate**			**Univariate**			**Multivariate**			**Univariate**			**Multivariate**		
Independent factors	*P value*	HR	95.0% CI	*P value*	HR	95.0% CI	*P value*	HR	95.0% CI	*P value*	HR	95.0% CI	*P value*	HR	95.0% CI	*P value*	HR	95.0% CI
Age	*0.875*	0.997	0.972-1.024				*0.645*	1.013	0.958-1.072				*0.877*	1.002	0.974-1.032			
ECOG-Performance status	*0.002*	1.433	1.151-1.869				*0.045*	1.776	1.012-3.115				*0.063*	1.297	0.986-1.704			
Gender (ref, female)	*0.073*	0.576	1.315-1.053				*0.018*	0.234	0.070-0.776				*0.086*	0.860	0.414-1.787			
Thrombus level (ref, renal vein)	*0.000*	1.472	1.185-1.830	*0.034*	1.275	1.0181.599							*0.179*	1.315	0.882-1.960			
Distant metastasis (ref, without metastasis)	*0.000*	4.115	2.020-8.384	*0.019*	2.548	1.168-5.560	*0.003*	9.673	2.113-44.27	*0.000*	34.01	5.368-215.6	*0.094*	1.996	0.888-4.4483			
Underwent surgery (ref, not performed)	*0.000*	0.144	0.069-0.303	*0.000*	0.239	0.108-0.532	*0.008*	0.180	0.051-0.636				*0.000*	0.105	0.036-0.305	*0.000*	0.061	0.016-0.230
Hemoglobin (g/dL)	*0.002*	0.807	0.704-0.926				*0.005*	0.645	0.477-0.873	*0.002*	0.500	0.323-0.775	*0.056*	0.863	0.742-1.004			
Serum albumin (g/dL)	*0.003*	0.487	0.306-0.778	*0.026*	0.849	0.736-0.980	*0.124*	0.537	0.244-1.186				*0.004*	0.408	0.221-0.756	*0.002*	0.250	0.104-0.600
eGFR (ml/min./1.73 m^2^)	*0.190*	0.989	0.974-1.005				*0.470*	0.989	0.961-1.019				*0.494*	0.999	0.995-1.002			
Choline esterase (U/L)	*0.052*	0.996	0.994-1.000	*0.023*	0.995	0.992-0.999	*0.156*	0.996	0.991-1.002				*0.002*	1.407	1.137-1.740	*0.053*	1.282	0.996-1.648
Neutrophil-lymphocyte ratio	*0.007*	1.297	1.072-1.571	*0.021*	1.250	1.034-1.513	*0.387*	1.204	0.791-1.835				*0.002*	1.407	1.137-1.740	*0.053*	1.282	0.993-1.648
Serum sodium (mEq/L)	*0.001*	0.814	0.721-0.921				*0.006*	0.774	0.644-0.931				*0.090*	0.862	0.723-1.023			
Correlated calcium (mg/dL)	*0.322*	1.143	0.877-1.491				*0.109*	1.982	0.858-4.579				*0.439*	0.854	0.572-1.274	*0.038*	0.598	0.368-0.973
Lactate dehydrogenase (LDH, IU/L)	*0.000*	1.004	1.002-1.006				*0.071*	1.004	1.001-1.008				*0.019*	1.003	1.001-1.006			
C-reactive protein (CRP, mg/dL)	*0.044*	1.046	1.001-1.093				*0.032*	1.083	1.007-1.165				*0.299*	1.029	0.975-1.0865			
Charlson comorbidity index	*0.000*	1.258	1.130-1.401	*0.006*	1.196	1.053-1.360	*0.002*	1.442	1.144-1.818				*0.022*	1.152	1.020-1.3007			
Molecular targeted agents (ref, not used)	*0.704*	1.148	0.561-2.351				*0.661*	0.711	0.155-3.275				*0.466*	1.362	0.593-3.124			

### RCC patients with tumor thrombus extending to the renal vein

Thirty-five of the 42 patients in the RV thrombus group received surgical management and 7 did not. None of these patients underwent metastasectomy, except for lymph node dissection. There were no significant differences between patients who received surgical management and those who did not in terms of age, sex, ECOG-PS, tumor histology, and blood biochemistry data. However, there were significant differences between these two groups in clinical T stage, lymph node involvement, presence of distant metastasis, tumor grade, Charlson comorbidity index, and administration of molecular targeted agents. In patients who received surgical management, the median blood loss was 744 g (range 10–3,221 g). Five patients with blood loss >1,500 g received blood transfusions. Two patients (6%) developed perioperative complications of Clavien grade ≥ III, and there were no deaths within 1 month of surgery. No patients required cardiopulmonary bypass during surgery. In the whole RV thrombus group, the estimated median survival time was “undefined”, and the estimated 5-year survival rate was 70%. In patients who received surgical management, the estimated median survival time was “undefined” and the estimated 5-year survival rate was 78%. In patients who did not receive surgical management, the estimated median survival time was 4.9 months and the estimated 5-year survival rate was 0% (Figure 3B, Table 2). In patients with distant metastasis at presentation, the estimated median survival time was 26 months and the estimated 5-year survival rate was 42%. In patients without distant metastasis at presentation, the estimated median survival time was “undefined” and the estimated 5-year survival rate was 90% (Figure 3C, Table 2). The independent prognostic factors identified by multivariate analysis using the Cox proportional hazards model in the RV thrombus group were presence of distant metastasis and hemoglobin concentration. Surgical management was not an independent prognostic factor in this group (Table 3).

### RCC patients with tumor thrombus extending to the IVC

Thirty of the 43 patients in the IVC thrombus group received surgical management and 13 did not. Ten of these patients underwent metastasectomy (adrenal, n = 1; bone, n = 1; lymph nodes, n = 2; liver, n = 1; liver, n = 2; IVC, n = 4) at the time of radical nephrectomy with thrombectomy. Four of these patients had positive surgical tumor margins, and these four all died within 3 years of surgery (median overall survival time: 26 months). There were significant differences between patients who received surgical management and those who did not in ECOG-PS, clinical T stage, lymph node involvement, tumor grade, serum sodium concentration, and Charlson comorbidity index. Other factors, including presence of distant metastasis, did not differ significantly between these two groups. In patients with thrombus level I, II, and III/IV who received surgical management, the median blood loss was 620 g (range 80–953 g), 1,255 g (range 810–5,100 g), and 3,397 g (range 2,454–13,800 g), respectively. Eighteen patients underwent blood transfusion because of blood loss >1,000 g or hypotension. Cardiopulmonary bypass was performed in three patients. Eight patients (19%) developed perioperative complications of Clavien grade ≥ III and two patients (6.7%) died within 1 month of surgery. There was no correlation between the level of IVC tumor thrombus and overall survival (Figure 3D). In the whole IVC thrombus group, the estimated median survival time was 24 months and the estimated 5-year survival rate was 23%. In patients who received surgical management, the estimated median survival time was 29 months and the estimated 5-year survival rate was 32%. In patients who did not receive surgical management, the estimated median survival time was 5.1 months and the estimated 5-year survival rate was 0% (Figure 3E, Table 2). In patients with distant metastasis at presentation, the estimated median survival time was 17 months and the estimated 5-year survival rate was 12%. In patients without distant metastasis at presentation, the estimated median survival time was “undefined” and the estimated 5-year survival rate was 90% (Figure 3F, Table 2). The independent prognostic factors identified by multivariate analysis using the Cox proportional hazards model in the IVC thrombus group were surgical management, serum albumin concentration, adjusted serum calcium concentration, and neutrophil-lymphocyte ratio (Table 3).

### Propensity score-matched analysis

Propensity score-matched analysis was used to compare overall survival between patients who received surgical management and those who did not. Conventionally recognized risk factors for survival including age, sex, ECOG-PS, level of tumor thrombus, presence of distant metastasis, and Charlson comorbidity index were controlled for using logistic analysis. Thirty pair-matched patients (15 patients who received surgical management and 15 who did not) were selected for analysis. The characteristics of these 30 patients are shown in Table 4. The only significant difference between patients who received surgical management and those who did not, including both matched and unmatched variables, was in serum sodium concentration.

**Table 4:** Characteristics of the patients included in the propensity score-matched analysis

**Propensity score matched patients**	**Surgery performed**	**Surgery not performed**	**P value**
<Matched parameters>			
n	15	15	
Age*	63 ± 11	63 ± 11	0.923
Gender (male/female)	9/6	9/6	1.000
ECOG-PS*	0.7 ± 1.2	0.9 ± 1.1	0.758
Presence of distant metastasis (M+)	13 (87%)	12 (80%)	0.624
Thrombus level			0.624
Renal vein	7	6	
I	1	2	
II	4	2	
III	2	3	
IV	1	2	
<Not matched parameters>			
Clinical TN			
cT3	15 (100%)	12 (80%)	0.068
cT4	0 (0%)	3 (20%)	
cN+	5 (33%)	10 (66%)	0.068
Hemoglobin (g/dL)*	11 ± 2.4	11 ± 3.2	0.857
Neutrophil-lymphocyte ratio*	3.2 ± 1.5	3.5 ± 1.2	0.636
Serum albumin (g/dL)*	3.8 ± 0.5	3.6 ± 0.6	0.331
Renal function (ml/min./1.73 m^2^)*	60 ± 16	58 ± 20	0.756
Lactate dehydrogenase (LDH, IU/L)*	200 ± 57	279 ± 201	0.160
Choline esterase (U/L)*	240 ± 110	188 ± 107	0.213
Serum sodium (mEq/L)*	141 ± 1.9	139 ± 3.0	0.028
Correlated Calcium (mg/dL)*	9.9 ± 1.0	9.9 ± 0.7	0.977
C-reactive protein (CRP, mg/dL)*	6.0 ± 7.3	5.3 ± 4.6	0.787
Charlson comorbidity index*	10 ± 1.5	10 ± 2.5	0.864
Molecular targeted argents	6 (40%)	6 (40%)	1.000
Deceased	10 (67%)	11 (73%)	0.690

*(Mean ± SD).

**Figure 4:**
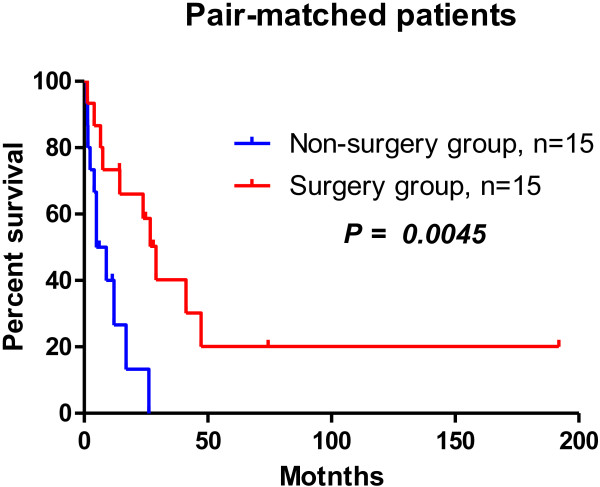
**Overall survival curves, estimated median survival times, and estimated 5-year survival rates in pair-matched patients.** Comparison of the 15 pairs of propensity score-matched patients suggests that surgical management may improve survival.

Among propensity score-matched patients, the overall survival time was significantly longer in those who received surgical management than those who did not. In patients who received surgical management, the estimated median survival time was 29 months and the estimated 5-year survival rate was 20%. In patients who did not receive surgical management, the estimated median survival time was 7 months and the estimated 5-year survival rate was 0% (Figure 4).

## Discussion

Aggressive surgical management with a hope of cure is currently the standard treatment for RCC patients with tumor thrombus extending to the RV and IVC, even though such surgery is challenging. In this study, the perioperative mortality rate in patients with IVC thrombus was 6.7%, which is significantly higher than the overall perioperative mortality rate of <0.1% in patients who undergo urological surgery at our institute. Although many studies reported benefits associated with surgical management in RCC patients with tumor thrombus extending to the RV and IVC [10,16-18], only a few studies compared outcomes between patients who underwent surgery and those who did not [8,11]. No randomized studies assessing the benefits of surgical management in RCC patients with a poor prognosis have been reported, because the decisions regarding surgical management of these patients depend on factors such as performance status, severe comorbidities, and the presence of metastasis. The largest reported series of RCC patients with renal vein and IVC thrombus, which included 1,122 patients with a median follow-up period of 24.7 months, found a median overall survival time of 33.8 months [19]. However, the role of surgical management in that series is unclear because outcomes were not compared between RCC patients with tumor thrombus who underwent surgery and those who did not. In this study, we used propensity score-matched analysis to retrospectively compare outcomes between RCC patients with renal vein and IVC thrombus who received surgical management and those who did not.

In all 85 patients studied, the following factors were identified as independent predictors of overall survival: thrombus level, presence of distant metastasis, surgical management, serum albumin concentration, serum choline esterase concentration, neutrophil-lymphocyte ratio, and Charlson comorbidity index.

This study found that patients who underwent radical nephrectomy with thrombectomy survived longer than those who received non-surgical management. However, the significance of this observation is limited by selection bias and the significant differences in variables between patients who received surgical management and those who did not. Patients who did not receive surgical management had a higher ECOG-PS and greater tumor burden, including unresectable disease and metastasis. The median survival times in patients who received surgical management and those who did not are comparable with those previously reported [8,11,18].

In the RV thrombus group, most patients (83%) received surgical management. Patients with thrombus that does not extend beyond the renal vein are more likely to have organ-confined disease and to be surgical candidates than patients with IVC thrombus. There were no significant differences between patients with thrombus extending to the renal vein who received surgical management and those who did not in terms of age and ECOG-PS, but there were significant differences between these two groups in organ-confined disease, presence of distant metastasis, tumor grade, Charlson comorbidity index, and administration of targeting agents. These findings suggest that RCC patients with more advanced disease were less likely to receive surgical management. The estimated median survival time was significantly longer in patients who received surgical management than in those who did not (“undefined” vs. 4.9 months). The estimated median survival times in this study are comparable with those previously reported [8,10]. Multivariate analysis of the RV thrombus group identified presence of distant metastasis and hemoglobin concentration as independent prognostic factors for overall survival. The estimated 5-year survival rate was 90% in patients with distant metastasis and 42% in patients without distant metastasis.

In the IVC thrombus group, 70% of patients received surgical management. There were no significant differences between patients who received surgical management and those who did not in terms of age, thrombus level, or presence of distant metastasis, but there were significant differences between these two groups in ECOG-PS, clinical T stage, clinical N stage, tumor grade, serum sodium concentration, and Charlson comorbidity index. These findings suggest that patients with advanced RCC and poor general status were more likely to receive non-surgical management. As in the RV thrombus group, the estimated median survival time was significantly longer in patients who received surgical management than those who did not (29 vs. 5.1 months, P < 0.0001). The estimated median survival times in this study are comparable with those previously reported [8,10,11,18]. Multivariate analysis of the IVC thrombus group identified surgical management, serum albumin concentration, neutrophil-lymphocyte ratio, and adjusted serum calcium concentration as independent prognostic factors for overall survival; but not thrombus level or presence of distant metastasis. The relationship between the level of IVC thrombus and prognosis is currently unclear [10,19-21]. Kim *et al.* reported that the level of tumor thrombus was not a predictor of cancer-specific survival on multivariate analysis (HR 0.95, P = 0.872) [20]. In contrast, Martinez-Salamanca *et al*. reported that tumor thrombus extending above the diaphragm (HR 2.10, P = 0.00), tumor diameter >7 cm, Furman grade, fat invasion, lymph node metastasis, and presence of distant metastasis were independent predictors of cancer-specific survival [19]. In this study, thrombus level of IVC was not identified as a significant predictor of overall survival. There was also no significant difference in overall survival between patients with suprahepatic IVC thrombus and those with infrahepatic IVC thrombus only (data not shown). This lack of association between thrombus level and survival may be due to the relatively small sample size.

The presence of distant metastasis has been reported to be a very powerful prognostic factor in RCC patients with tumor thrombus [10]. Lambert *et al.* studied RCC patients with venous tumor thrombus, and found a 5-year cancer-specific survival rate of 10.0% in patients with distant metastasis and 60.3% in those without distant metastasis [22]. Although the presence of distant metastasis was not identified as an independent prognostic factor in this study, the estimated 5-year overall survival rate was significantly lower in patients with distant metastasis than those without (12% vs. 54%, P = 0.0883).

We used propensity score-matched analysis to control for patient characteristics when comparing overall survival between patients who received surgical management and those who did not. The variables used for the logistic analysis were conventionally recognized risk factors for survival, including age, sex, ECOG-PS, level of tumor thrombus, presence of distant metastasis, and Charlson comorbidity index. Table 4 shows the matched and unmatched variables in patients who received surgical management and those who did not. Comparison of the 15 pairs of propensity score-matched patients suggests that surgical management may improve survival. The estimated median survival time was four times longer in patients who received surgical management than those who did not (P = 0.0045).

The findings of this study and of previous studies [3,8-10,17,18,23] suggest that the upper limit of the 5-year survival rate in RCC patients with venous thrombus who receive surgical management may be 50–60%. Because these patients have a high rate of metastasis at presentation, optimal systemic therapy, such as neoadjuvant therapy using molecular-targeting agents, is important. The first report of neoadjuvant sunitinib therapy in an RCC patient with IVC thrombus described a significant down-staging of the thrombus and reduction of the extent of surgery required [24]. Subsequent studies reported significant reduction in tumor thrombus after preoperative sunitinib [25] or sorafenib therapy [16]. These data suggest that neoadjuvant therapy can downsize thrombus with minimal invasiveness, thereby increasing surgical resectability. However, some studies did not report good results after neoadjuvant therapy [23,26]. In 2011, Cost *et al.* reported that targeted therapy resulted in only a 12% cytoreductive effect and a high rate of progression. The usefulness of neoadjuvant targeted therapy therefore remains unclear.

This study has several limitations, including the small sample size and the inclusion of only patients treated at a single institution. These factors make it difficult to control for selection bias and patient characteristics. However, an advantage of this study is the use of propensity score matching between patients who received surgical management and those who did not. The results show an overall survival benefit for patients who received surgical management. Randomized trials including larger numbers of patients from multiple institutions are necessary to clarify the benefits of surgical management in patients with RCC with venous thrombus.

## Conclusions

Surgical management may improve the prognosis of RCC patients with thrombus extending to the renal vein and IVC.

## Abbreviations

CT: Computed tomography; ECOG-PS: Eastern cooperative oncology group performance status; IVC: Inferior vena cava; RCC: Renal cell carcinoma; RV: Renal vein.

## Competing interests

The authors declare that they have no competing interests.

## Authors’ contributions

SH drafted the manuscript. TK participated in drafting the manuscript. TY, IH, HM, TN, MO, KH, DN, TT, YT, and YH performed the clinical follow-up and collected data. CO was responsible for the concept and design of the study, the interpretation of data, and critical revision of the manuscript. All authors read and approved the final manuscript.

## Authors’ information

SH: lecturer; TY: lecturer; IH: resident in training; HM: postgraduate student; TN: postgraduate student; MO: postgraduate student; KH: postgraduate student; DN: postgraduate student; TT: postgraduate student; YT: postgraduate student; YH: associate professor; TK: associate professor; CO: professor and chairman, Department of Urology, Hirosaki Graduate School of Medicine.

## Pre-publication history

The pre-publication history for this paper can be accessed here:

http://www.biomedcentral.com/1471-2490/13/47/prepub
